# Interventions for zoster-associated pain: A retrospective study based on the clinical database

**DOI:** 10.3389/fneur.2022.1056171

**Published:** 2022-11-24

**Authors:** Lu Chen, Jun Li, Hui Liu, Pingliang Yang, Yunxia Zuo, Ling Ye

**Affiliations:** ^1^Department of Pain Management, West China Hospital, Sichuan University, Chengdu, China; ^2^Department of Anesthesiology, The First Affiliated Hospital of Chengdu Medical College, Chengdu, China; ^3^Department of Anesthesiology, West China Hospital, Sichuan University, Chengdu, China

**Keywords:** herpes zoster, postherpetic neuralgia, database system, pain, quality of life

## Abstract

**Background:**

Herpes zoster (HZ)-associated pain can lead to severe pain and reduced quality of life. Exploring effective treatment and the risk factors of zoster-associated pain has become important.

**Methods:**

Interventions including nerve block, radiofrequency, and thermocoagulation were used for zoster-associated pain. The data of 131 patients with HZ and 230 patients with postherpetic neuralgia (PHN) were collected at baseline, 2 weeks, 3, 6, and 12 months after the intervention. Visual analog scale (VAS) and Brief Pain Inventory (BPI) scores at different time points were analyzed by two-way repeated measures ANOVA with Group as the between-subject variable (different pain durations and areas), Time as the within-subject variable (baseline, 2 weeks, 3 months, 6 months, and 12 months), and Group × Time as the two-way interaction. Besides, the effective rate, adverse effects, and medication were also recorded. The risk factors of PHN were analyzed by logistic regression.

**Results:**

A total of 25 (19.08%) patients with HZ continued to have pain in the initially affected area after 3 months. The VAS scores and the BPI quality of life scores of patients with HZ-associated pain were significantly reduced from baseline to 2 weeks, 3, 6, and 12 months after the interventions. There was no significant difference in VAS and BPI scores in different pain areas and pain durations. No significant Group × Time interaction was observed. Age, diabetes mellitus, and immune-related diseases were risk factors of PHN.

**Conclusion:**

Interventions could significantly improve the pain degree and life quality of patients with zoster-associated pain, and the positive effect of intervention did not change with pain duration and area. Advanced age, diabetes, and immune-related diseases are risk factors of PHN.

## Introduction

Herpes zoster (HZ) is caused by varicella herpes virus infection with an annual incidence of 2–5 per 1,000, which might lead to severe pain (1, 2). As the most common complication of HZ, postherpetic neuralgia (PHN) is defined as pain in the lesion area 3 months after the shingles (3). Once PHN is developed, the treatment is extremely difficult and less effective. Patients with zoster-associated pain suffered from severe physical, occupational, social, and psychological disabilities (4). Therefore, exploring effective treatment and risk factors of zoster-associated pain has become particularly important.

At present, treatment for zoster-associated pain includes medication and interventional treatment. Medication includes gabapentin, pregabalin, nonsteroidal anti-inflammatory drugs (NSAIDs), opioids, antidepressants, and topical treatment. Interventional treatment includes epidural or paravertebral nerve blocks, pulsed radiofrequency (PRF), and thermocoagulation. Nerve block is recommended, while oral medication is ineffective, but evidence of PRF and thermocoagulation for first-line treatment is limited. Furthermore, the risk of PHN may be related to some clinical features of HZ, including prodromal pain, degree of pain, and rash density (5). The incidence of PHN and the difficulty of treatment increase with age (6, 7). Some studies have found that patients with risk factors who did not receive effective intervention are more likely to develop PHN (8–10). However, there are considerable differences among the current studies, and the evidence on risk factors is limited. Therefore, this study was designed to investigate the efficacy of interventions for zoster-associated pain and risk factors of PHN.

## Methods

### Design and participants

This retrospective study was registered at ChiCTR.org.cn (ChiCTR1800015561, date of registration: April 2018) and was approved by the Ethics Committee of West China Hospital, Sichuan University, Chengdu, China (No. 2018 [25], date of approval: 7 February 2018). The data of patients diagnosed with HZ and PHN were extracted from the department of pain management of West China Hospital of Sichuan University from September 2017 to September 2018. In this study, HZ included the acute stage (1 month after rash onset) and the subacute stage after the acute phase and before PHN was diagnosed (1–3 months), while PHN was diagnosed as pain that persists for more than 3 months after rash onset. An experienced pain physician evaluated neuropathic pain according to the NeuPSIG grading system and assessed the pain intensity by the visual analog scale (VAS) (11). Inclusion criteria were as follows: (1) over 18 years old; (2) diagnosed with HZ or PHN; (3) VAS >3 points before treatment; and (4) accompanied by neuropathic pain in the lesion area. Exclusion criteria were as follows: (1) multiple locations involved; (2) with severe cardiovascular disease, pulmonary disease, or abnormal liver and kidney function; (3) severe pain caused by other diseases; (4) alcohol and drug abuse; and (5) with mental disorders and cannot obtain informed consent.

### Data collection based on database system

A database was set up for this study in the clinical research data management system of West China Hospital. The researchers could independently design the clinical case report form (CRF) and filter the information, including the general information, course of the disease, location of pain, clinical intervention, pain degree, quality of life, and other information. Besides, the database provides the features of automatic enrollment, follow-up reminders, authority allocation, and other functions. The researchers collected relevant data as follows:

(1) Clinical data of patients, including demographic data, disease-related data, and medical history.
(2) Pain was measured by VAS and pain relief rate. VAS is a 10-cm straight line that only marks the pain at both ends. One end is 0, indicating no pain; the other end is 10, indicating severe pain. Patients are asked to mark the level of pain on the line. The pain relief rate is ranked as apparent effect (pain relief ≥50% after treatment), effective (pain relief 30–50%), and ineffective (pain relief <30%). The remission rate was calculated (number of apparent and effective effect patients/total number of patients).
(3) Quality of life was measured by the Brief Pain Inventory (BPI) quality of life part, which contains 7 dimensions, including general activity, mood, walking ability, normal work, relation with other people, sleep, and enjoyment of life. Each dimension was measured by scores that ranged from 0 to 10, with higher scores indicating a greater impact on the quality of life.
(4) Adverse reactions include dizziness, nausea, vomiting, puncture injury (pneumothorax, bleeding, and nerve injury), infection, limb movement disorder, incontinence, and respiratory disease.
(5) Medication: The types and doses of drugs.
(6) Follow-up time: baseline, 2 weeks, 3, 6, and 12 months after the intervention.

### Interventional treatment

#### HZ

Patients with HZ usually received different nerve block methods (epidural block, semilunar ganglion block, peripheral nerve block, stellate nerve block, etc.). Patients in the subacute stage (1–3 months) received CT-guided PRF nerve regulation twice with an interval of 1 week. Two or three intervertebral foramina of adjacent segments were located and punctured with a radiofrequency catheter under the guidance of CT. After reconfirming the target by sensory-motor electrical stimulation, the PRF mode was administered at 42°C for 15 min, and then 1 ml of the mixture of compound betamethasone and lidocaine was injected for its anti-inflammation and analgesic effect.

#### PHN

For patients with PHN in the trigeminal nerve distribution region, physicians usually punctured the radiofrequency needle focusing on the foramina of the lesion side under CT guidance. After reaching the foramina, a mixture of 40 mg methylprednisolone and 1 ml of 0.5% ropivacaine was injected into the trigeminal ganglion. If there was no adverse reaction in 5 min, 0.5 ml of 0.5% adriamycin was injected. When PHN occurred in the area of the trunk, CT-guided radiofrequency thermocoagulation and analgesia injection were administered. After locating two or three intervertebral foramina of adjacent segments, the physician punctured the radiofrequency catheter under the guidance of CT and reconfirmed the target by sensory-motor electrical stimulation. Then, standard radiofrequency model has two cycles at 80°C, 2 min per cycle was performed, and after that, 1 ml of the mixture of compound betamethasone and lidocaine was injected into the intervertebral foramen and ipsilateral epidural cavity under CT guidance. If no adverse reactions were observed in 5 min, the physician could inject 1 ml of 0.33% or 0.5% adriamycin at each segment injection. In the area of limbs, radiofrequency was changed from standard radiofrequency to PRF to avoid long-time continuous radiofrequency affecting the motor neurons associated with limb movement, which might influence the quality of life. Besides, 2–3 ml of analgesic fluid was injected in each segment instead of adriamycin.

### Statistical analysis

The SPSS version 23.0 software was used for statistical analysis. Descriptive statistics were used to summarize the baseline characteristics of the participants. The measurement data were expressed as mean ± standard deviation, and the ranked data were represented as rate and ratio. VAS and BPI scores in patients with HZ and PHN at different time points were analyzed by two-way repeated measures ANOVA with Group as the between-subject variable (different pain durations and areas), Time as the within-subject variable (baseline, 2 weeks, 3, 6, and 12 months), and Group × Time as the two-way interaction. Linear regression was used to analyze the correlation between age, gender, course, location, complications, and VAS score. Logistic regression analysis was performed in patients with HZ to explore the risk factors of PHN. The dependent variable was whether patients with HZ developed PHN. The independent variables included age (<65 years or ≥65 years), diabetes, immune-related disease, and osteoporosis. These binary variables were assigned “yes” to 1 and “no” to 0 in logistic regression analysis. P < 0.05 was considered statistically significant.

## Results

### Basic characteristics

The data of 131 patients with HZ and 230 patients with PHN were extracted from the database system from September 2017 to September 2018, with an overall sample loss rate of 6.36%. The basic characteristics of patients are shown in Table 1.

**Table 1:** The basic characteristics of patients.

**Characteristic**		**HZ(*n =* 131)**	**PHN(*n =* 230)**
Age (y)		61.86 ± 12.39	67.90 ± 10.20
Sex, *n* (%)	Male	65(49.6%)	119(51.7%)
	Female	66(50.4%)	111(48.3%)
Course, *n* (%)	<3 months	131 (100%)	
	4–6 months		93(40.4%)
	7–12 months		71(30.9%)
	>12 months		66(28.7%)
Area, *n* (%)	Facial (Trigeminal nerve)	16(12.2%)	27(11.7%)
	Neck and upper limbs (C2-8)	26(19.8%)	37(16.1%)
	Trunk (T1-12)	79(60.4%)	147(63.9%)
	Hip and legs (L1-5; S1-5)	10(7.6%)	19(8.3%)
Complications, *n* (%)	Hypertension	28(21.4%)	37(16.1%)
	Diabetes	19(14.5%)	43(18.7%)
	Osteoporosis	11(8.4%)	23(10%)
	Immune related diseases	19(14.5%)	19(8.3%)
	COPD	4(3.1%)	15(6.5%)

HZ, herpes zoster; PHN, postherpetic neuralgia; COPD, chronic obstructive pulmonary disease.

### Pain

**Figure 1:**
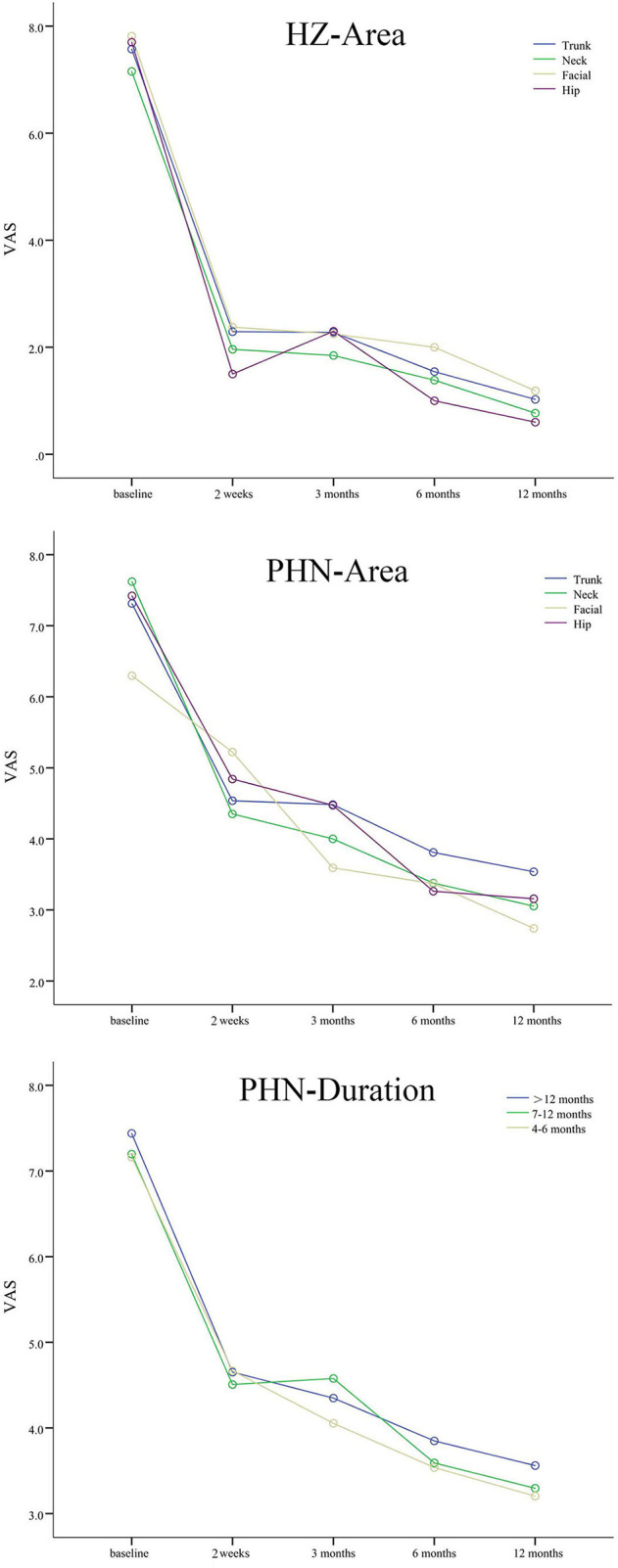
The VAS scores of patients with HZ and PHN. Different colored lines mean different pain areas and durations. The VAS scores decreased significantly from baseline to 2 weeks, 3, 6, and 12 months after interventions in patients with different pain durations and areas.

A total of 25 (19.08%) patients with HZ continued to have pain in the initially affected area after 3 months. In Table 2 and Figure 1, the VAS decreased significantly from baseline to 2 weeks, 3, 6, and 12 months after the intervention in patients with HZ and PHN (*P* < 0.001). There was no significant difference in VAS in different pain areas (*P* = 0.346 in HZ; *P* = 0.347 in PHN) and pain durations (*P* = 0.555 in PHN). No significant Group × Time interaction was observed, which indicated that different groups followed similar trends of VAS over time and the intervention had a positive effect on patients with different pain durations and areas.

**Table 2:** Two-way repeated measures ANOVA for between group and time interactions of VAS scores in patients with HZ and PHN.

**Disease**	**Group**	**Number**	**Baseline**	**2 weeks**	**3 months**	**6 months**	**12 months**		**F**	***P*** **-value**
HZ	Trunk	79	7.57 ± 1.55	2.29 ± 1.22	2.28 ± 1.79	1.54 ± 1.71	1.03 ± 1.50	Group	1.115	0.346
	Neck	26	7.15 ± 2.20	1.96 ± 1.11	1.85 ± 1.52	1.38 ± 1.50	0.77 ± 1.03	Time	355.076	<0.001
	Facial	16	7.81 ± 1.68	2.38 ± 0.89	2.25 ± 1.69	2.00 ± 1.55	1.19 ± 1.22	Group*Time	0.476	0.876
	Hip	10	7.70 ± 0.95	1.50 ± 0.97	2.30 ± 1.83	1.00 ± 1.15	0.60 ± 0.84			
PHN	Trunk	147	7.31 ± 1.51	4.54 ± 2.21	4.48 ± 1.79	3.81 ± 1.96	3.54 ± 2.18	Group	1.106	0.347
	Neck	37	7.62 ± 1.67	4.35 ± 2.42	4.00 ± 1.51	3.38 ± 1.74	3.05 ± 1.86	Time	90.084	<0.001
	Facial	27	6.30 ± 2.16	5.22 ± 1.99	3.59 ± 1.69	3.37 ± 1.86	2.74 ± 1.83	Group*Time	2.407	0.068
	Hip	19	7.42 ± 1.57	4.84 ± 2.24	4.47 ± 1.81	3.26 ± 1.91	3.16 ± 2.81			
	>12 months	66	7.44 ± 1.62	4.65 ± 2.38	4.35 ± 1.83	3.85 ± 2.05	3.56 ± 2.13	Group	0.590	0.555
	7–12 months	71	7.20 ± 1.60	4.51 ± 2.17	4.58 ± 1.64	3.59 ± 1.67	3.30 ± 2.19	Time	169.970	<0.001
	4–6 months	93	7.16 ± 1.73	4.67 ± 2.17	4.05 ± 1.77	3.54 ± 2.00	3.20 ± 2.16	Group*Time	0.707	0.657

HZ, herpes zoster; PHN, postherpetic neuralgia; VAS, visual analog scale; ANOVA, analysis of variance.

Besides, there were significant differences between baseline and 2 weeks, 3, 6, and 12 months after the intervention in patients with HZ and PHN in pain relief (*P* < 0.05) (Table 3).

**Table 3:** Pain relief in patients with HZ and PHN.

**Pain relief**		**Time points**			
		**2 weeks**	**3 months**	**6 months**	**12 months**
Apparent	HZ	128(97.7%)	120(91.6%)	121(92.4%)	127(96.9%)
	PHN	95(41.3%)	85(36.9%)	137(59.6%)	139(60.5%)
Effective	HZ	1(0.8%)	5(3.8%)	5(3.8%)	1(0.8%)
	PHN	27(11.7%)	64(27.8%)	41(17.8%)	39(16.9%)
Non-effective	HZ	2(1.5%)	6(4.6%)	5(3.8%)	3(2.3%)
	PHN	108(47%)	81(35.2%)	52(22.6%)	52(22.6%)
Remission rate	HZ	129(98.5%)	125(95.4%)	126(96.2%)	128(97.7%)
	PHN	122(53%)	149(64.7%)	178(77.3%)	178(77.3%)

HZ, herpes zoster; PHN, postherpetic neuralgia.

### Quality of life

**Figure 2:**
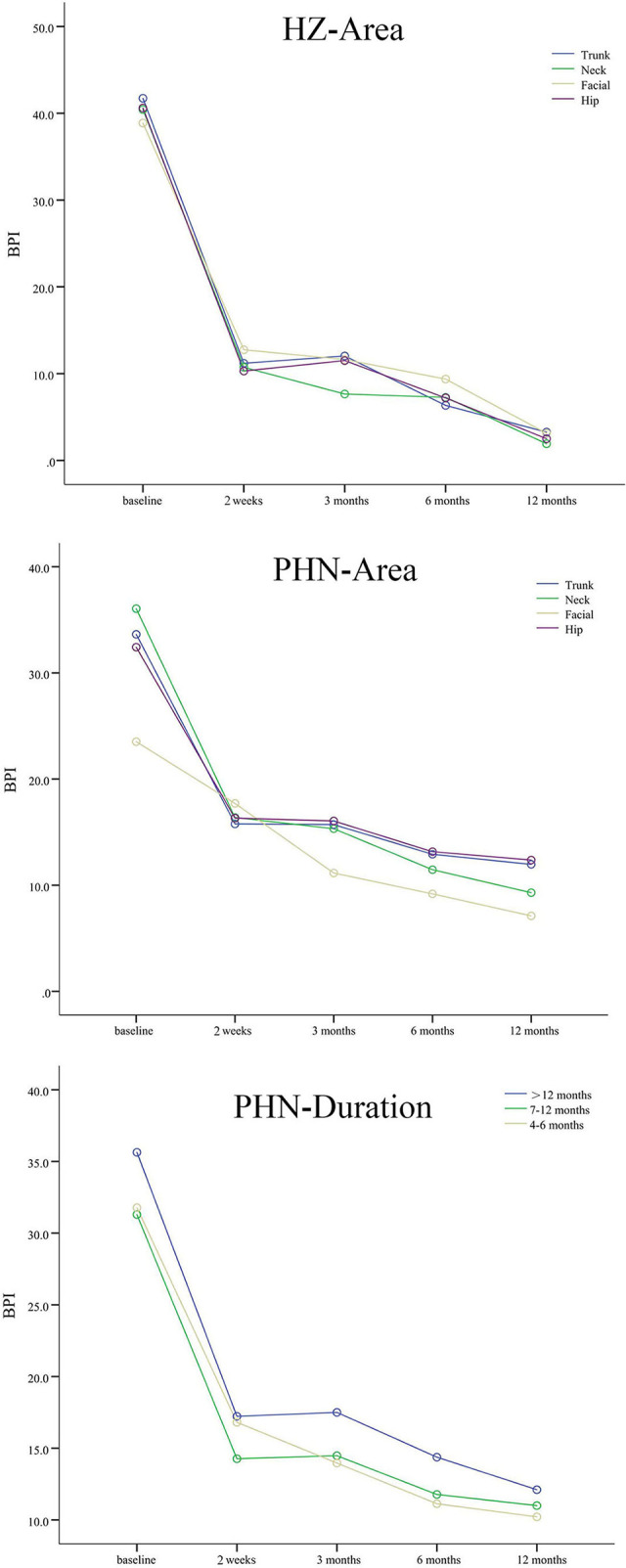
The BPI scores of patients with HZ and PHN. Different colored lines mean different pain areas and durations. The BPI scores decreased significantly from baseline to 2 weeks, 3, 6, and 12 months after interventions in patients with different pain durations and areas.

The data of 7 dimensions of BPI at baseline and 2 weeks, 3, 6, and 12 months after the intervention in HZ and PHN groups are shown in Table 4. The total scores of BPI decreased significantly from baseline to 2 weeks, 3, 6, and 12 months after the intervention in patients with HZ and PHN, which confirmed the effect of interventions on quality of life (*P* < 0.001). There was no significant difference in BPI in different pain areas (*P* = 0.609 in HZ; *P* = 0.147 in PHN) and pain durations (*P* = 0.117 in PHN). There was no significant Group × Time interaction, suggesting that different groups followed similar trends of BPI over time, and the positive effect of intervention did not change with pain duration and area (Figure 2 and Table 5).

**Table 4:** Dimensions of BPI scores of patients with HZ and PHN.

**Dimensions**		**Time points**				
		**Baseline**	**2 weeks**	**3 months**	**6 months**	**12 months**
General activity	HZ	5.98 ± 1.74	1.56 ± 1.19	1.73 ± 1.54	1.22 ± 1.32	0.53 ± 1.25
	PHN	4.19 ± 2.88	2.03 ± 2.12	2.05 ± 2.04	1.87 ± 1.32	1.53 ± 1.25
Mood	HZ	6.11 ± 2.48	2.02 ± 1.03	1.85 ± 1.69	1.24 ± 1.31	0.64 ± 1.11
	PHN	5.15 ± 2.55	2.45 ± 2.01	2.57 ± 2.06	2.14 ± 2.04	1.64 ± 1.11
Walking ability	HZ	4.18 ± 1.96	1.18 ± 1.15	1.06 ± 1.15	0.70 ± 0.97	0.15 ± 0.39
	PHN	3.48 ± 2.66	2.23 ± 2.12	2.51 ± 1.76	1.24 ± 0.97	1.10 ± 0.79
Normal work	HZ	5.46 ± 2.26	1.42 ± 1.21	1.32 ± 1.32	0.77 ± 1.03	0.16 ± 0.47
	PHN	4.87 ± 2.48	2.80 ± 2.05	2.33 ± 2.05	1.95 ± 1.13	1.80 ± 2.15
Relation with others	HZ	5.24 ± 1.91	1.44 ± 1.06	1.27 ± 1.31	0.89 ± 1.13	0.29 ± 0.67
	PHN	3.29 ± 2.69	1.63 ± 1.76	2.57 ± 1.77	1.25 ± 0.62	1.10 ± 1.55
Sleep	HZ	6.69 ± 1.86	1.79 ± 1.09	1.84 ± 1.54	0.96 ± 1.20	0.50 ± 0.92
	PHN	5.44 ± 2.58	1.79 ± 2.33	2.97 ± 2.20	2.36 ± 2.14	2.15 ± 2.14
Enjoy of life	HZ	7.34 ± 2.02	1.85 ± 1.24	2.02 ± 1.59	1.20 ± 1.34	0.66 ± 1.02
	PHN	5.34 ± 2.67	2.84 ± 1.24	2.65 ± 2.14	2.13 ± 2.04	1.95 ± 1.93

HZ, herpes zoster; PHN, postherpetic neuralgia; BPI, brief pain inventory.

**Table 5:** Two-way repeated measures ANOVA for between group and time interactions of BPI scores in patients with HZ and PHN.

**Disease**	**Group**	**Number**	**baseline**	**2 weeks**	**3 months**	**6 months**	**12 months**		***F***	***P*** **-value**
HZ	Trunk	79	41.72 ± 9.06	11.19 ± 5.39	12.04 ± 10.33	6.34 ± 7.51	3.27 ± 5.29	Group	0.612	0.609
	Neck	26	40.42 ± 11.94	10.73 ± 6.31	7.65 ± 7.13	7.27 ± 7.18	1.92 ± 3.78	Time	335.868	<0.001
	Facial	16	38.88 ± 11.02	12.75 ± 9.14	11.62 ± 5.86	9.38 ± 9.16	3.12 ± 4.40	Group*Time	60.870	0.899
	Hip	10	40.60 ± 7.55	10.30 ± 6.38	11.50 ± 8.64	7.20 ± 7.44	2.50 ± 3.98			
PHN	Trunk	147	33.63 ± 14.53	15.78 ± 11.33	15.71 ± 10.91	12.91 ± 12.08	11.97 ± 12.53	Group	1.805	0.147
	Neck	37	36.05 ± 16.87	16.38 ± 11.31	15.32 ± 12.34	11.46 ± 10.65	9.30 ± 8.86	Time	77.983	<0.001
	Facial	27	23.52 ± 14.43	17.70 ± 14.31	11.15 ± 10.37	9.19 ± 10.31	7.11 ± 8.16	Group*Time	2.635	0.051
	Hip	19	32.42 ± 17.50	16.32 ± 7.80	16.05 ± 12.83	13.16 ± 13.45	12.37 ± 11.76			
	>12 months	66	35.64 ± 14.25	17.23 ± 11.32	17.50 ± 12.09	14.38 ± 12.54	12.11 ± 12.30	Group	2.163	0.117
	7-12 months	71	31.30 ± 16.99	14.27 ± 12.00	14.48 ± 11.06	11.77 ± 12.55	11.00 ± 12.24	Time	163.967	<0.001
	4-6 months	93	31.77 ± 14.97	16.81 ± 10.97	13.97 ± 10.70	11.13 ± 10.47	10.22 ± 10.53	Group*Time	0.039	0.962

HZ, herpes zoster; PHN, postherpetic neuralgia; BPI, brief pain inventory; ANOVA, analysis of variance.

### Medication

The most commonly used drugs for patients with HZ were pregabalin, oxycodone and acetaminophen, and tramadol. After the intervention of nerve block and PRF, the drug use gradually decreased with the extension of time, the number of combined drug users decreased significantly, and the decrease was obvious at 3, 6, and 12 months after the intervention.

Pregabalin, gabapentin, oxycodone and acetaminophen, and lidocaine cream were the most commonly used drugs in patients with PHN. After clinical interventions, the use of those drugs gradually decreased with time, as well as the combined drug. At each follow-up time point, the use of pregabalin, gabapentin, and oxycodone and acetaminophen was still higher than that of other drugs, and the use of lidocaine cream and anti-anxiety and anti-depressants decreased significantly (Table 6).

**Table 6:** Medication for patients with HZ and PHN.

**Medication**		**Time points**				
		**Baseline**	**2 weeks**	**3 months**	**6 months**	**12 months**
NSAIDS	HZ	12(9.2%)	2(1.5%)	0(0%)	0(0%)	0(0%)
	PHN	18(7.8%)	3(1.3%)	0(0%)	0(0%)	0(0%)
Pregabalin	HZ	90(68.7%)	66(50.4%)	22(16.8%)	16(15.5%)	5(3.8%)
	PHN	138(60%)	128(55.7%)	118(51.3%)	96(41.7%)	76(33%)
Gabapentin	HZ	8(6.1%)	12(9.2%)	9(6.9%)	2(1.9%)	0(0%)
	PHN	92(40%)	85(36.9%)	92(40%)	90(39.1%)	75(32.6%)
Carbamazepine/Oxcarbazepine	HZ	4(3.1%)	4(3.1%)	0(0%)	0(0%)	0(0%)
	PHN	20(9.6%)	12(5.2%)	5(2.2%)	0(0%)	0(0%)
Oxycodone and Acetaminophen	HZ	80(62.6%)	21(16%)	12(9.2%)	5(4.9%)	2(1.5%)
	PHN	158(68.7%)	128(56%)	92(40%)	75(32.6%)	52(22.6%)
Tramadol	HZ	7(5.34%)	4(3.1%)	0(0%)	0(0%)	0(0%)
	PHN	34(14.8%)	15(6.5%)	8(3.5%)	0(0%)	0(0%)
Lidocaine cream	PHN	112(48.7%)	60(26%)	42(18.3%)	28(12.2%)	19(8.3%)
Anti-anxiety and anti-depressants	PHN	47(16.5%)	40(17.4%)	28(12.2%)	12(5.4%)	5(2.2%)
Combined medication	HZ	118(89.3%)	87(66.4%)	12(9.2%)	5(6.8%)	2(1.5%)
	PHN	210(92.1%)	184(80.4%)	112(48.7%)	85(36.9%)	65(28.2%)

HZ, herpes zoster; PHN, postherpetic neuralgia.

### Adverse reaction

The total incidence of adverse reactions for patients with HZ was 9.2%. There were 8 cases of dizziness and nausea, 2 cases of somnolence, and 2 cases of transient pain. No serious adverse reactions occurred.

A total of 18 (7.8%) cases of adverse reaction were reported after intervention in patients with PHN, including 10 cases of dizziness and nausea, 3 cases of palpitation, 4 cases of transient pain after the invasive treatment, and 1 case of urinary disturbance which was relieved after catheterization.

### Correlation and regression analysis

Linear regression analysis showed that VAS score in patients with HZ (*N* = 131) and PHN (*n* = 230) was positively correlated with age (HZ: *r* = 0.238, P < 0.001, PHN: *r* = 0.151, *P* = 0.023). Notably, 19.08% of patients with HZ developed PHN after the intervention (25/131). Logistic regression analysis found that the risk factors of PHN were advanced age (odds ratio [OR] = 1.17, 95%CI: 1.09–1.26, *p* = 0.000), diabetes (OR = 5.04, 95%CI: 1.30–19.51, *p* = 0.019), and immune-related diseases (OR = 18.53, 95%CI: 2.88–118.93, *p* = 0.002). Osteoporosis was not the risk factor of PHN (OR = 0.081, 95%CI: 0.006–1.061, *P* = 0.056).

## Discussion

In 2019 S2k guidelines for the diagnosis and treatment of HZ and PHN (12), systemic analgesics are recommended for acute HZ-associated pain including antiepileptic agents (gabapentin and pregabalin), NSAIDs, and opioids. Patients with PHN can also be added with topical treatment such as capsaicin 8% patch and lidocaine 5% patch. Besides, epidural or paravertebral local anesthetic and steroid nerve blocks could be an option for HZ-associated pain, while oral medication is ineffective. Although the evidence of PRF for PHN was considered low quality in 2013 Interventional management of neuropathic pain: NeuPSIG recommendations (13), a systematic review suggested PRF as second-line treatment for PHN in 2019 based on the relevant research in recent years (14). Besides, interventional treatments such as PRF and thermocoagulation are recommended in 2016 Chinese expert consensus on the diagnosis and treatment of PHN (15).

### Efficacy of intervention

In this study, the data of a total of 131 patients with HZ and 230 patients with PHN were extracted. The VAS scores of patients with HZ were significantly reduced from severe pain (VAS ≥ 7) to mild pain (VAS ≤ 3) after interventions, which was similar to a previous study (16). Patients with significant pain relief (pain relief ≥50%) account for more than 90% of participants, and the reduction in pain was positively correlated with the improvement in quality of life. In the early stage of HZ, medication and nerve block treatment played an important role in pain control, which might be related to relieving the inflammation, promoting blood circulation, and breaking the vicious circle of pain. In the subacute stage (1–3 months), the effect of epidural nerve block on pain relief was decreased compared with that in the acute stage (17). Besides, PRF is more effective than the epidural block in the subacute stage of HZ by reducing the nerve sensitivity which was caused by the continuous transmission of pain signals (18, 19), which is similar to our results.

After active clinical interventions, 25 (19.08%) patients developed PHN, which was less than a previous report (6). We also found that VAS scores and the frequency of pain episodes were lower than baseline. The number of patients with apparent pain relief at each follow-up time point was significantly increased, but the remission rate at 6 months and 12 months was not significantly increased (*P* > 0.05). With the relief of pain, the total scores of BPI decreased after the intervention, and 7 dimensions including general activity, mood, walking ability, normal work, relation with other people, sleep, and enjoyment of life were improved.

In some pain areas and durations such as trunk, hip, and 7–12 months group, postoperative VAS scores and BPI quality of life scores decreased 2 weeks after the intervention, with a recurrent increase or stabilization in 3 months, and then gradually improved from 6 to 12 months, which might be related to the mechanism of radiofrequency thermocoagulation, adriamycin, and glucocorticoid injection. Radiofrequency relieved the pain by destroying nerve fibers, especially unmyelinated nerve fibers (20). Adriamycin could cause swelling and necrosis in neurons, decrease the Nissl bodies, and increase the pain threshold 2 weeks after injected into the dorsal root ganglion (21). Moreover, glucocorticoid was also injected to reduce inflammation which was caused by nerve damage (22), and the pain was relieved significantly, especially in the early postoperative period (23).

In addition, the decline in the BPI score was greater than the VAS score, which suggested that the improvement in quality of life is superior to the relief of pain. Long-term chronic pain, especially neuropathic pain, is prone to cognitive behavioral disorders such as anxiety and depression. At present, a large number of studies have shown that emotional state has a great impact on chronic pain. Therefore, psychological and behavioral interventions can help patients to accept the impact of pain and establish self-management awareness of chronic pain (24). At the same time, invasive therapy can greatly reduce the use of analgesic drugs; thus, the reduction of side effects also improved the quality of life.

### Safety

Adriamycin is used to treat PHN because of its destructive effect on the nerve by reversing axonal transport. As basic studies have confirmed that a high concentration of adriamycin may damage motor neurons (25) and excessive dosage of adriamycin will lead to more risk of cardiac toxicity, there are clear clinical limits on the upper dose of adriamycin (26). In the treatment of PHN, 0.5% of adriamycin is rarely seen to impair motor function and sensory disorders, the therapeutic dose of adriamycin is much lower than the toxic dose, and it is administered by local injection that does not directly enter the blood circulation, so the risk of cardiac toxicity is significantly reduced. In addition, all interventional therapies are guided by CT, which can accurately puncture to the target location, reducing the side effects caused by repeated punctures and overdosed drugs. In this study, the incidence of adverse effects caused by intervention measures was 9.2% and 7.8% in the HZ and PHN groups. The most common complications were nausea, vomiting, and dizziness, which were of low degree and were improved after symptomatic and supportive treatment. One patient in the PHN group had urinary retention and symptoms were relieved after catheterization, which might be related to the entry of adriamycin into the subarachnoid space through the nerve root sleeve in the vicinity of the cauda equina nerve at the lesion stage. Although the current clinical intervention therapy for HZ and PHN was relatively safe in this study, thermocoagulation is an irreversible and destructive procedure, which could potentially have complications such as numbness or even decreased muscle strength, especially since neuropathic pain is already caused by a lesion of the nervous system. As a result, this destructive treatment should be chosen carefully.

### Stratified analysis

We found that the degree of pain and the quality of life are less affected in the facial area of patients with PHN, which may be due to the relatively small range of skin lesions in this area and avoiding pain triggers easily.

Considering the repair process of neuropathic injury, PHN was suggested to be classified according to the duration of the disease including stage 1 (3 months), stage 2 (3–6 months), and stage 3 (>6 months), which might be useful for stratified analysis (27). In this study, we found that VAS and BPI scores of patients with a course over 12 months were higher than other groups, so we choose the time points of grouping including 4–6, 7–12, and >12 months in stratified analysis. There was no statistical difference in VAS and BPI scores in different pain durations and areas, which confirmed the positive effect of interventions on patients with HZ and PHN. Current studies have shown that neuroplasticity may be the pathological basis of PHN, in which peripheral axonal injury and degeneration of sensory neurons and spinal dorsal horn neurons are typical characteristics (28). As a result, we suggest treating patients with HZ or PHN as soon as possible.

### Risk factors

In a large retrospective study containing 119,413 patients with HZ, the risk factors of PHN included age, women, immune-related diseases, asthma, diabetes, smoking, underweight, and obesity (29). In linear regression analysis, we found that with the increase of age, the pain degree of HZ-associated pain was more severe. It was also found that the average age of the patients with PHN was 67.9 years. Among them, 44% were patients aged 65–80 years, and 40% were older than 80 years. Patients with diabetes mellitus accounted for 40%, and patients with immune-related diseases (rheumatic immune diseases such as tumors) accounted for 20%. Furthermore, by using logistic regression, we found that age, diabetes, and immune-related diseases were risk factors of PHN. The risk of PHN for elderly patients with HZ was 1.18 times higher than others; for patients with diabetes mellitus, it was five times higher than others; and for patients with immune-related diseases, it was 18 times higher than others. Previous studies have also proved that age is one of the main reasons leading to the decline of immune function (30). Diabetes usually causes abnormal metabolism of glycolipids and amino acids in the body, leading to changes in immune function, which mostly affects the metabolic characteristics of the body (31). Therefore, it is suggested the detection of abnormal immune function and metabolism, and the exploration of specific markers related to metabolism may also assist with the screening, prediction, and early intervention of PHN.

### Limitation

However, there are some limitations in this study due to the retrospective characteristics of the analysis. First of all, the missing placebo arm might weaken the credibility of the results. Besides, the individual dosage of analgesic medication may have a greater impact on therapeutic effect than the interventions. There are some missing values of the individual dosage of analgesic medication; as a result, we only analyzed the proportion not the dosage of analgesic medication, which could be analyzed in the future study by improving the database system. Furthermore, the comparison between the interventions might not be thorough enough, which could be improved in further study.

## Conclusion

Interventions could significantly improve the pain degree and life quality of patients with zoster-associated pain, and the positive effect of intervention did not change with pain duration and area. Besides, advanced age, diabetes, and immune-related diseases are risk factors that could influence the occurrence of PHN.

## Data availability statement

The raw data supporting the conclusions of this article will be made available by the authors, without undue reservation.

## Ethics statement

The study was approved by the Ethics Committee of West China Hospital, Sichuan University, Chengdu, China (No. 2018 [25], date of approval: 2 February 2018). Written informed consent from the patients/participants or patients/participants' legal guardian/next of kin was not required to participate in this study in accordance with the national legislation and the institutional requirements.

## Author contributions

LC and JL were the major contributors to writing the manuscript. JL designed the study and collected the data with LC. PY and LY analyzed the data. HL and YZ revised the manuscript. All authors read and approved the final manuscript.

## Funding

This study was supported by grant 2022NSFSC0710 from the Science and Technology Department of Sichuan Province and grant 2022-YF05-01343-SN from the Chengdu Science and Technology Bureau. The funders did not participate in any aspect of the trial, including design, data collection, analysis, or interpretation.

## Conflict of interest

The authors declare that the research was conducted in the absence of any commercial or financial relationships that could be construed as a potential conflict of interest.

## Publisher's note

All claims expressed in this article are solely those of the authors and do not necessarily represent those of their affiliated organizations, or those of the publisher, the editors and the reviewers. Any product that may be evaluated in this article, or claim that may be made by its manufacturer, is not guaranteed or endorsed by the publisher.
